# Supplementary material for: Incorporating the image formation process into deep learning improves network performance
Source: Nat Methods. 2022 Oct 31;19(11):1427–37. doi: 10.1038/s41592-022-01652-7 (PMC9636023; doi:10.1038/s41592-022-01652-7)
Supplement: Supplementary file 1 — Supplementary Notes 1 and 2, Figs. 1–8 and Tables 1–4. [file 41592_2022_1652_MOESM1_ESM.pdf]

nature methods

Article

https://doi.org/10.1038/s41592-022-01652-7

Incorporating the image formation process into deep learning improves network performance

In the format provided by the authors and unedited

Supplementary information

Incorporating the image formation process into deep learning improves network performance

Yue Li[1], Yijun Su[2,3,#], Min Guo[2], Xiaofei Han[2], Jiamin Liu[3], Harshad D. Vishwasrao[3], Xuesong Li[2,#], Ryan Christensen[2,#], Titas Sengupta[4], Mark W. Moyle[5], Ivan Rey-Suarez[6], Jiji Chen[3], Arpita Upadhyaya[6,7], Ted B. Usdin[8], Daniel Colón-Ramos[9,10], Huafeng Liu[1,11,*], Yicong Wu[2,*], Hari Shroff[2,3,10,#]

1. State Key Laboratory of Modern Optical Instrumentation, College of Optical Science and Engineering, Zhejiang University, Hangzhou, Zhejiang, 310027, China
2. Laboratory of High Resolution Optical Imaging, National Institute of Biomedical Imaging and Bioengineering, National Institutes of Health, Bethesda, MD 20892, USA
3. Advanced Imaging and Microscopy Resource, National Institutes of Health, Bethesda, MD 20892, USA
4. Lewis-Sigler Institute for Integrative Genomics, Princeton University, Princeton, NJ 08540, USA
5. Department of Biology, Brigham Young University-Idaho, Rexburg, ID 83440, USA
6. Institute for Physical Science and Technology, University of Maryland, College Park, MD 20742
7. Department of Physics, University of Maryland, College Park, MD 20742
8. Systems Neuroscience Imaging Resource, National Institute of Mental Health, National Institutes of Health, Bethesda, MD 20892, USA
9. Wu Tsai Institute, Department of Neuroscience and Department of Cell Biology, Yale University School of Medicine, New Haven, CT 06536, USA
10. MBL Fellows Program, Marine Biological Laboratory, Woods Hole, MA 02543, USA
11. Intelligent Optical and Photonics Research Center, Jiaxing Research Institute, Zhejiang University, Jiaxing, Zhejiang, 314000, China

*Correspondence to Yicong Wu, yicong.wu@nih.gov and Huafeng Liu, liuhf@zju.edu.cn
# Current affiliation: Janelia Research Campus, Howard Hughes Medical Institute, Ashburn, VA, 20147 USA

The supplementary information includes **Supplementary Notes 1, 2** (page 2-8), **Supplementary Figures 1-8** (page 9-17), and **Supplementary Tables 1-4** (page 18-24).

Supplementary Note 1: RLN vs. Deep-URL and USRNet

Like RLN, there are other neural networks based on algorithm unrolling[1] (also called 'deep unfolding'[2]). For example, Deep-URL[3] and USRNet[4] also incorporate model-based formulae into the learning-based method and try to bridge the gap between learning-based methods and model-based methods. However, RLN is quite different from these two methods, and its unique architecture enables several advantages.

USRNet is a single end-to-end trained network designed to handle super-resolution tasks with different scale factors, blur kernels, and noise levels. As we summarize in **Figure SN1.1**, the architecture of USRNet includes three core components: data modules, prior modules, and a hyper-parameter module. The USRNet alternates between the data module and the prior module using K deconvolution iterations, where K was empirically set to 8 to balance speed and accuracy. Unlike RLN, which does not need to specify an iteration number, here K is a user-determined tunable parameter that must be determined in advance for each application – like classic deconvolution. The data module contains no trainable parameters, but requires a scale factor (another user-determined parameter) and a blurring kernel as input. The prior module integrates residual blocks into a U-Net architecture and uses the result of the data module and the noise level map as input. The hyper-parameter module consists of three fully connected layers and controls the outputs of the data module and prior module by varying the scale factor and noise level.

This architecture leads to clear disadvantages compared to RLN. First, USRNet is only designed for 2D images. It would be challenging to extend USRNet to 3D, which would require more GPU memory and computational overhead. Since USRNet is heavily reliant on FFT and inverse FFT operations in each data module, using a prior module based on the UNet architecture will introduce considerably more computational burden if using a 3D UNet. For example, the content-aware image restoration network (CARE) architecture (based on a 3D UNet and including two downsample levels) is 4x slower than RLN (**Fig. 1c** in the main text). USRNet requires three downsample levels, suggesting the prior module in 3D USRNet will be even more computationally expensive than CARE, and thus much worse than RLN. Second as mentioned above, USRNet requires a tunable parameter K to provide reasonable results. Third, the blurring kernel must be known and fed into the network, limiting the usage of USRNet to applications in which the PSF is constant over the field of view ('spatially invariant PSF'). Last, the computational burden even for 2D applications is high, for example 2 days were required to train a 2D network model using 4 Nvidia Tesla V100 GPUs, for the parameters used in the published work (training iterations ~2 x 10^5, training images size of 96 x 96, and batch size set to 128). By contrast, RLN performs 3D image deconvolution without needing to explicitly provide (or tune) either a blurring kernel or an iteration number, thus achieving rapid training and processing times much faster than USRNet on 3D data (e.g., ~2-3h using a single Nvidia GeForce GTX 1080 Ti GPU, training iteration number is ~2 x 10^4, training volumes sized of 64 x 64 x 64 with batch size set to 4).

Deep-URL is designed for 2D blind deconvolution, aiming to predict both the deconvolved image and the blurring kernel. Deep-URL (**Figure SN1.2**) is motivated by the Richardson-Lucy algorithm like RLN (**Figure SN1.3**), but Deep-URL defines the number of network layers exactly analogously to the deconvolution iteration number K (e.g., 2, 5 in the published work) in the

Richardson-Lucy formula at iteration k $(k = 1, 2, \dots. K)$:

$$H^{k+1} = \sigma\left(\mathrm{ReLU}\left(\left[\frac{y}{\mathrm{ReLU}\left(x^k * W_H^k\right)}\right] * x^{k\dagger}\right) \times W_H^k\right)$$

$$x^{k+1} = \sigma\left(\mathrm{ReLU}\left(\left[\frac{y}{\mathrm{ReLU}\left(W_x^k * H^{k+1}\right)}\right] * H^{k+1\dagger}\right) \times W_x^k\right).$$

The learning parameters W_H^k and W_x^k in the *k*-th layer (deconvolution iteration) have the same size as the blurring kernel H^{k+1} and input image x^{k+1}. Although the computational burden is never specified in the paper, we suspect the large size of these parameters combined with the necessary convolutions very likely contributes to a long training time, perhaps infeasibly long for 3D applications. Another concern is the application of Deep-URL in tasks that require a spatially varying PSF, as Deep-URL outputs only a single PSF. In addition, Deep-URL was only demonstrated on simulated data; its performance on experimental data is unknown. RLN differs from Deep-URL in the following aspects: (1) for most fluorescence microscopes, the point spread function can be measured or modelled, and thus RLN does not need to predict the blur kernel explicitly, which simplifies network architecture; (2) RLN was motivated by our improved Richardson-Lucy algorithm, i.e., using an unmatched backprojector[5], and can achieve resolution-limited results with only 1 deconvolution iteration so there is no need to specify or tune an deconvolution iteration number *K*; (3) RLN makes use of many small [3 x 3 x 3] convolution kernels to perform learning, thereby rapidly and effectively performing 3D deconvolution.

In summary, we emphasize that the architecture of RLN is unique compared to USRNet and Deep-URL, and that this unique architecture provides notable advantages including extension to 3D imaging, the absence of a need to specify an explicit deconvolution iteration number in the network design, and considerably less computational burden than the other two networks, despite handling larger (3D) data. In addition, RLN can handle images with spatially variant blurring (**Extended Data Fig. 4g**), which would challenge USRNet and Deep-URL, since USRNet requires a fixed blurring kernel as input and Deep-URL predicts only a single blurring kernel.

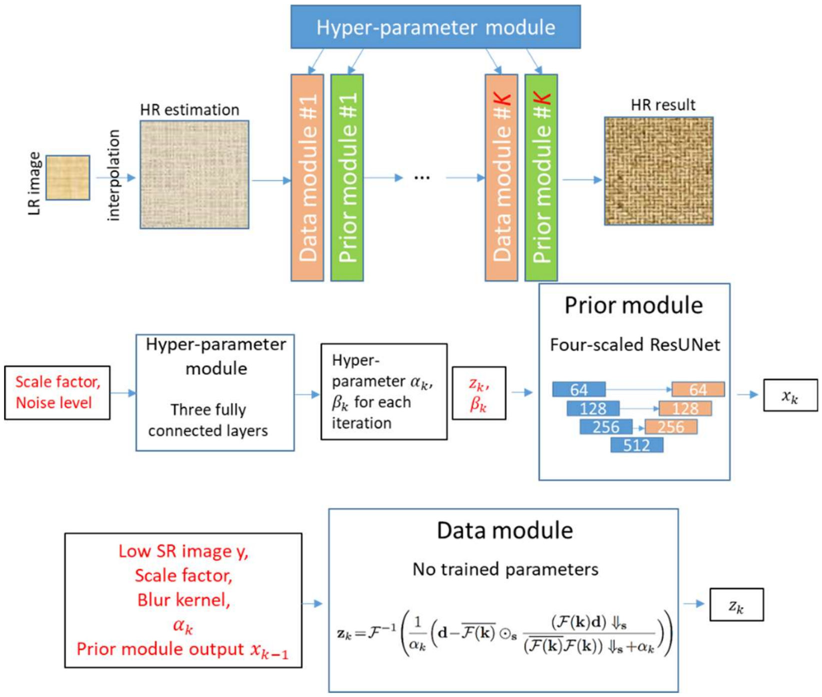

Figure SN1.1, The overall architecture of USRNet with the data module, prior module, and hyper-parameter module emphasized. In addition to the typical parameters required for deep learning (e.g., learning rate, batch size, decay rate, etc.), this architecture also requires the following tunable parameters (red): the deconvolution iteration number K for constructing the data and prior module, the blurring kernel, noise level, and scale factor. k = 1, 2, …, K represent intermediate iterations.

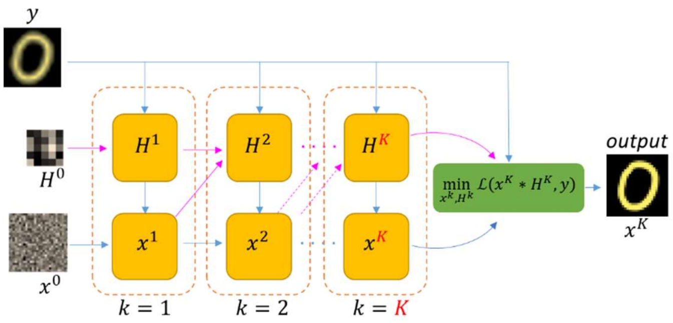

Figure SN1.2, The architecture of Deep-URL for model-aware blind deconvolution. Given a blurred image y and initial estimates of the clean image x^0 and blurring kernel H^0, the model updates x^k and H^k. The deconvolution iteration number K (red) must be chosen as it is used to determine how many layers are needed. K = 1, 2,…, K represent intermediate iterations.

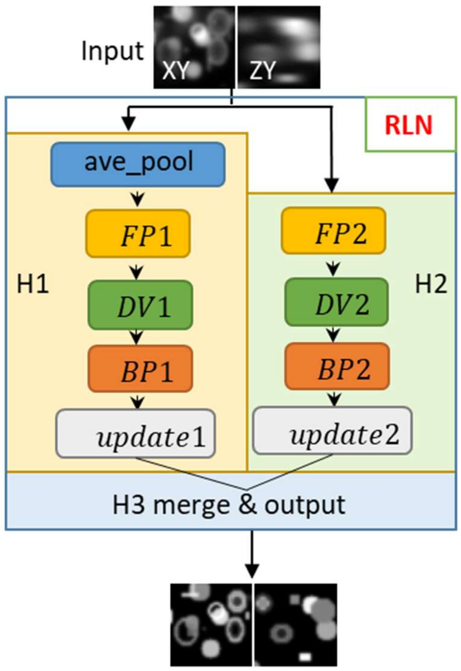

Figure SN1.3, The architecture of RLN for 3D deconvolution, consisting of three parts: down-scale estimation starting from the average-pooled input image, H1; original-scale estimation starting with original-scale input image, H2; and merging/fine-tuning, H3. H1 and H2 are inspired by the RL deconvolution update formula, which mimic the unmatched forward/back projector steps. H1 is used to increase the field of view and decrease the computational burden. H2 is used to provide information to assist H1. RLN only needs the learning-based parameters typical for any data-driven network; there are no model parameters to adjust.

Supplementary Note 2: RLN's interpretability and generalizability

Interpretability refers to the extent of a human's ability to understand the model, but it appears difficult to reach consensus on the exact meaning of the term[6, 7]. For example, some researchers explore post-hoc explanations for models, while others try to explore the interplay between the internal components / machinery of a model. RLN was motivated by, and is based on, Richardson-lucy deconvolution with unmatched backprojectors[5]. As the convolution operation plays key roles in both classic deconvolution algorithms and convolutional neural networks (CNN), RLN uses the convolutional layers of a CNN to replace the traditional convolution operation, solving the unmatched forward/backward projector design problem by combining data-driven training with an architecture that mimics the underlying RLD model. From this starting point, RLN can be interpreted as a projector-design algorithm and the role of different convolutional layers in RLN also can be explained.

In addition to showing the intermediate outputs after applying the forward projector *FP* and backward projector *BP* (e.g., **Fig. 1b**, **Extended Data Fig. 2**, and **Supplementary Figs. 2, 3**), here we further investigate all intermediate RLN outputs and attempt to link them to specific steps in RLD (**Figure SN2.1**), finding that H1 and H2 modules display both data-driven learning and behavior characteristic of RLD, while H3 is purely data-driven. As shown, the *FP* submodule provides gradually smoother features, the *DV* step enhances dim signals, the final results of *BP* provide the update factor, and resolution is enhanced after the update step. It is difficult to explain what the first few layers of *BP* (images with red borders) are doing, perhaps because there is no obvious link to RLD, but it appears that the last layers of BP in both H1 and H2 provide update factors that enhance contrast for fine features (e.g., edges).

Despite efforts to explain why networks do or do not generalize, there still does not appear to be a completely satisfactory explanation[8, 9]. RLN's generalization capability may be at least partially explained by the following: (1) although the network does not directly learn the PSF kernel used in forward and backward projection steps, the summed effect of the convolutional layers mimic an effective PSF analogous to that used in RLD, perhaps explaining the robustness to different types of data (as shown in **Figs. 3-5** in the main text, **Extended Data Figs. 9, 10**, and **Supplementary Figs. 3, 7, 8**); (2) the RLD formula embedded in the network structure acts to regularize training, helping to guide non-content-based feature learning; (3) the number of learning parameters in RLN is much less than in CARE and RCAN, which might reduce over-fitting.

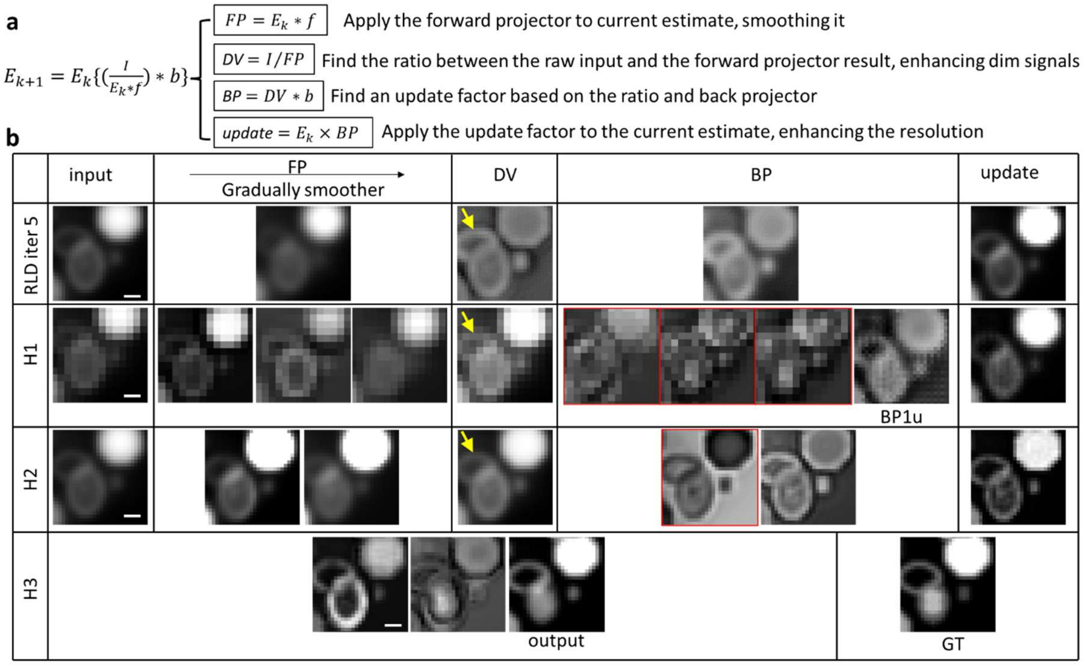

Figure SN2.1, Further links between RLN and RLD. a) The decomposition of RLD and the function of each step, illustrated with synthetic mixed structures. b) Comparisons between RLD and RLN at each intermediate output. Five iterations of RLD are shown (top row), compared with all intermediate feature maps generated by RLN (other rows), to illustrate where RLN does and does not agree with RLD. H1 and H2 display both data-driven behavior and more interpretable output analogous to RLD. The FP submodule provides gradually smoother features, the DV step enhances dim signals (yellow arrows), the final results of BP provide the update factor and resolution is enhanced after the update step. The first few layers of BP are not easy to understand because of the learning-based characteristic of convolutional layers (indicated by the red borders), but the last layer of BP provides an update factor that enhances contrast for fine features. H3 is based purely on data-driven learning and is used to merge the output of H1 and H2 part. Scalebar: 10 pixels.

References

1. Monga, V., Li, Y. & Eldar, Y.C. Algorithm Unrolling: Interpretable, Efficient Deep Learning for Signal and Image Processing. *IEEE Signal Processing Magazine* **38**, 18-44 (2021).
2. Balatsoukas-Stimming, A. & Studer, C. Deep Unfolding for Communications Systems: A Survey and Some New Directions. *2019 IEEE International Workshop on Signal Processing Systems (SiPS)*, 266-271 (2019)
3. Agarwal, C., Khobahi, S., Bose, A., Soltanalian, M. & Schonfeld, D. Deep-URL: A Model-Aware Approach to Blind Deconvolution Based on Deep Unfolded Richardson-Lucy Network. *2020 IEEE International Conference on Image Processing (ICIP)*, 3299-3303 (2020)
4. Zhang, K., Gool, L.V. & Timofte, R. Deep Unfolding Network for Image Super-Resolution. *2020 IEEE/CVF Conference on Computer Vision and Pattern Recognition (CVPR)*, 3214-3223 (2020)
5. Guo, M. et al. Rapid image deconvolution and multiview fusion for optical microscopy. *Nature*

Biotechnology **38**, 1337-1346 (2020).
6. Zhang, Y., Tiňo, P., Leonardis, A. & Tang, K. A Survey on Neural Network Interpretability. *IEEE Transactions on Emerging Topics in Computational Intelligence* **5**, 726-742 (2021).
7. Fan, F.L., Xiong, J., Li, M. & Wang, G. On Interpretability of Artificial Neural Networks: A Survey. *IEEE Transactions on Radiation and Plasma Medical Sciences* **5**, 741-760 (2021).
8. Zhang, C.Y., Bengio, S., Hardt, M., Recht, B. & Vinyals, O. Understanding Deep Learning (Still) Requires Rethinking Generalization. *Communications of the Acm* **64**, 107-115 (2021).
9. Chatterjee, S. & Zielinski, P. On the Generalization Mystery in Deep Learning. *arXiv e-prints* **https://arxiv.org/pdf/2203.10036v1.pdf** (2022).

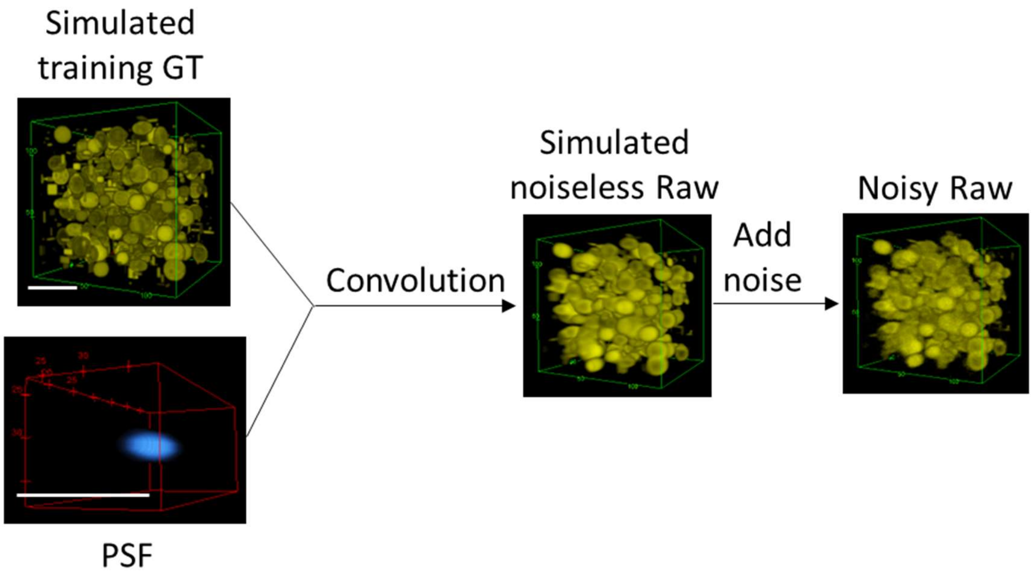

Supplementary Fig. 1, The training simulated data generation process. Simulated training ground truth (GT) consists of dots, solid spheres, and ellipsoidal surfaces. The noiseless raw data is generated by convolving the ground truth data with the PSF (enlarged at left for clarity). Scale bars: 5 µm.

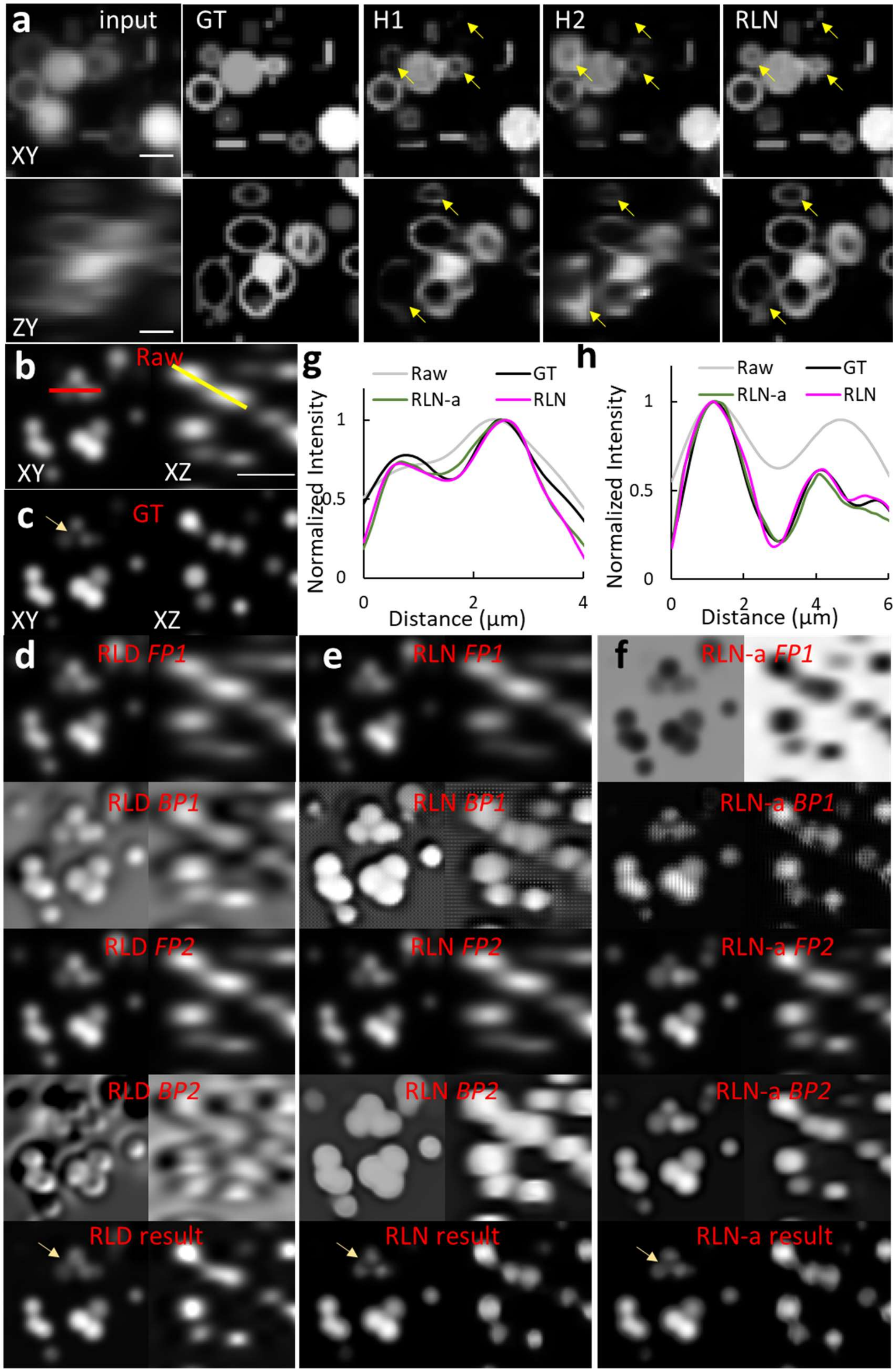

Supplementary Fig. 2, RLN outperforms H1 alone, H2 alone, and RLN-a. a) Comparing networks using only H1, only H2, or RLN on synthetic mixed structures (conventional testing). Lateral (top) and axial (bottom) views are shown. The combination of H1 and H2 provide better

performance than using H1 or H2 alone (yellow arrows). Quantitative analysis confirms this visual impression: H1 alone, PSNR 26.4 ± 0.5, SSIM 0.87 ± 0.03; H2 alone, PSNR 22.4 ± 0.4, SSIM 0.74 ± 0.01; RLN, PSNR 30.2 ± 0.3, SSIM 0.93 ± 0.01, N = 12 volumes. **b)** Raw input of simulated beads, which is used to compare RLD, RLN, and RLN-a performance (using training data from synthetic mixed structures). Lateral (left) and axial (right) views are shown in each case. **c)** Ground truth. **d-f)** Intermediate output of **d)** RLD, **e)** RLN, and **f)** RLN-a. RLN and RLN-a models were trained with the phantom objects consisting of dots, solid spheres, and ellipsoidal surfaces. RLN and RLN-a: *FP1*, *BP1*, *FP2*, *BP2* are the steps in H1 and H2; RLD: *FP1* and *BP1* are the outputs after forward projection and backward projection at iteration 1, and *FP2*, *BP2* at iteration 10. Dim details are better restored with RLN than RLD and RLD-a (yellow arrows). RLN shows better recovery (SSIM 0.97, PSNR 35.7) than RLN-a (SSIM 0.94, PSNR 34.0). Also, the intermediate output of RLN, appear visually closer to the intermediate steps of RLD, particularly the *FP1* step. These results suggest that the additional network structure in RLN (i.e., the *DV* and *update* steps) aids in network generalizability. **g, h**) Line profiles along the red and yellow lines in the XY and ZY views in **b),** showing RLN is closer to the ground truth than RLD and RLN-a. Scale bar: 2 µm in **a**, others are 5 µm.

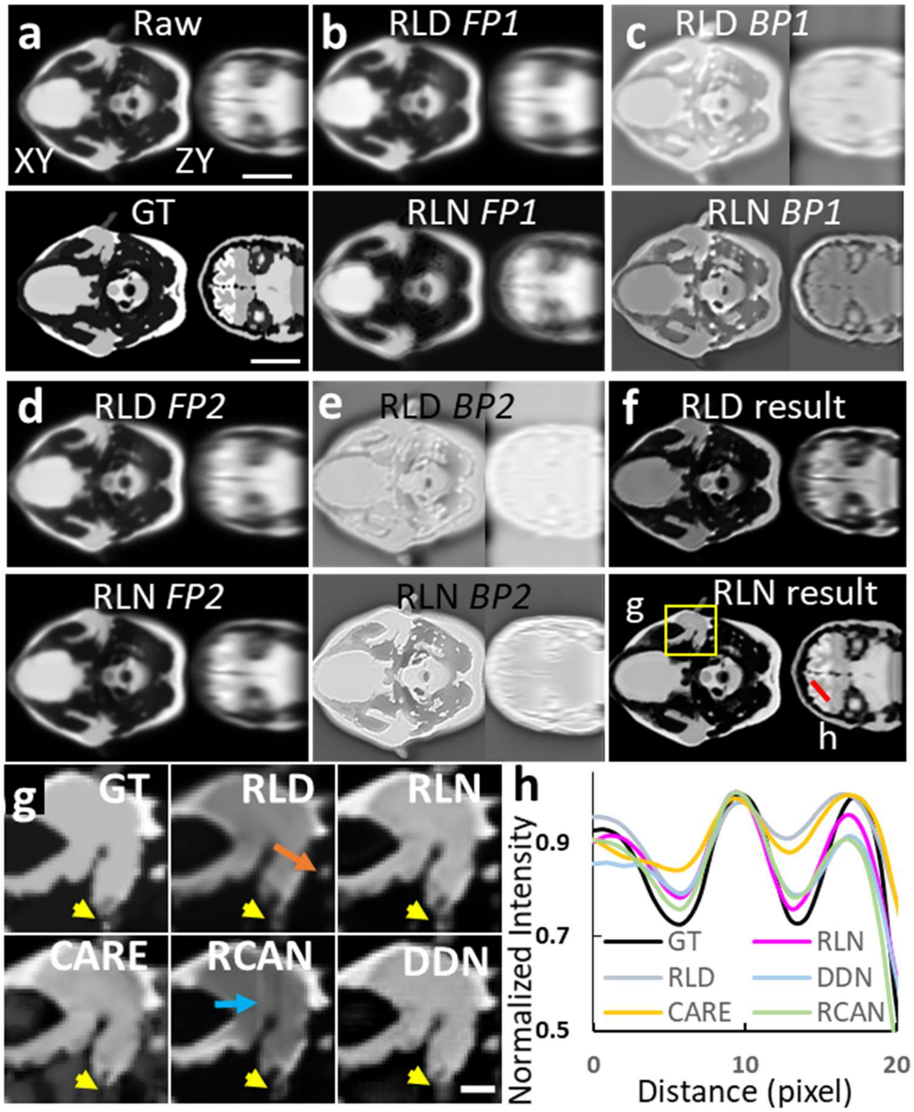

Supplementary Fig. 3, **Insight into the intermediate stages of RLN and generalization of RLN as assessed on human brain phantom. a)** Blurry raw input (top row) and ground truth (GT, bottom row). **b-e)** Intermediate output of RLD (top row) and RLN (bottom row) at *FP1* **(b)**, *BP1* **(c)**, *FP2* **(d)**, and *BP2* **(e)**. RLN: *FP1*, *BP1*, *FP2*, *BP2* are the steps in H1 and H2; RLD: *FP1* and *BP1* are the forward projection and backward projection at iteration 1, and *FP2*, *BP2* at iteration 20. Similarities in output between RLN and RLD indicates connections between RLN and RLD. **f)** Results of RLD (top row) and RLN (bottom row). The RLN result is noticeably closer to the ground truth (SSIM 0.89, PSNR 24.4) than RLD (SSIM 0.72, PSNR 16.9). Lateral (left) and axial (right) views are shown in **a-f)**. **g)** Magnified view of the yellow rectangle in **f)**, comparing the ground truth, RLD and the predictions from RLN, CARE, RCAN and DDN, with arrows highlighting features showing RLN provides better restoration than the other methods, see also **Fig. 1g** for quantification. **h)** Line profile along the red line in **f)**, showing RLN is closer to the ground truth than RLD and other networks. All models were trained with the phantom objects consisting of dots, solid spheres, and ellipsoidal surfaces. See also **Fig. 1f**. Scale bars: **a-f)** 50 pixels**, g)** 10 pixels.

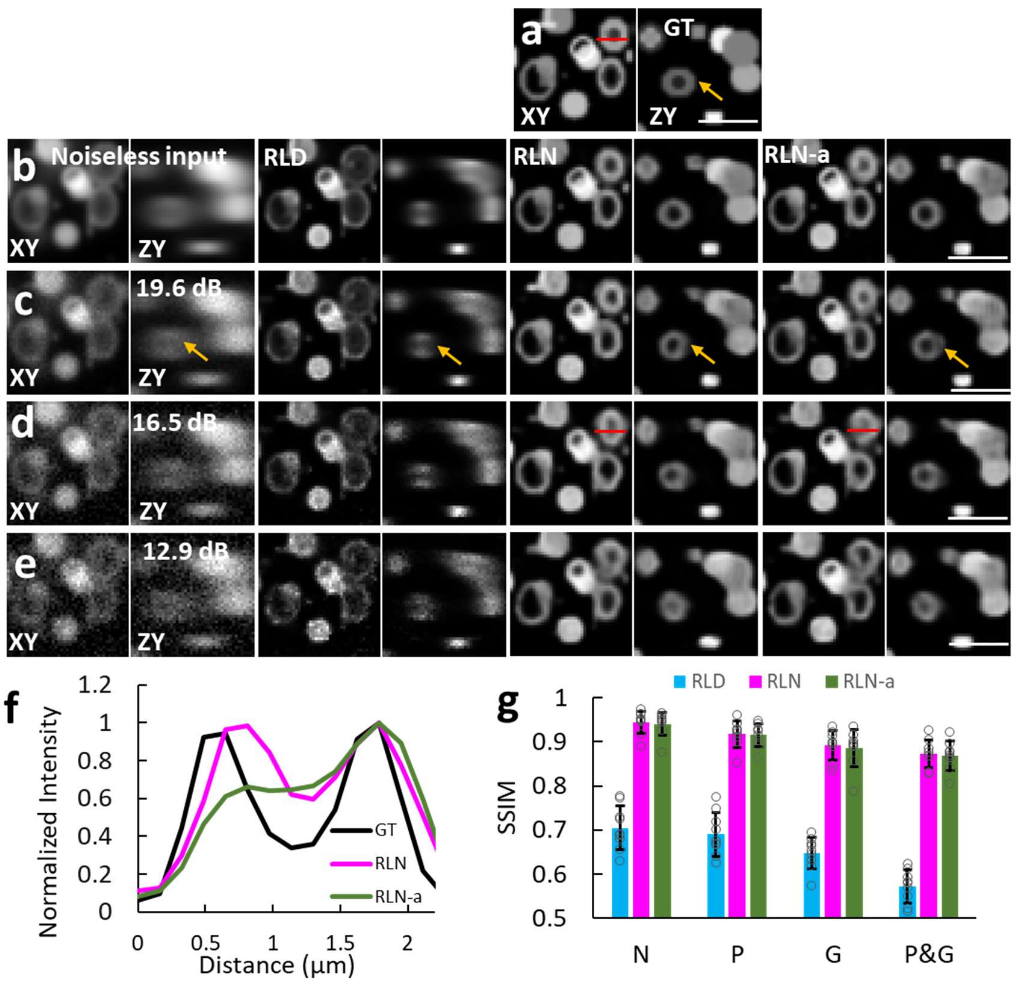

Supplementary Fig. 4, Deconvolution quality as a function of input SNR, comparing RLD, RLN and RLN-a trained with synthetic mixed structures dataset with corresponding SNR. a) Ground truth. **b-e)** Left to right columns show output of RLD, RLN, and RLN-a in lateral (left) and axial (right) views as a function of **b)** no noise (N); **c)** Poisson noise (P); **d)** Gaussian noise (G); **e**) mixed Poisson and Gaussian noise (P&G). **f)** Line profiles of the red lines in **a)**, **d)** showing that RLN prediction is closer to ground truth than RLN-a. **g)** SSIM and PSNR values of RLD, RLN, and RLN-a as a function of different noise types. In all cases, SSIM and PSNR decrease as noise increases, and RLN and RLN-a show considerably higher SSIM and PSNR than RLD at all noise levels. Individual values (open circles), means, and standard deviations from N=9 volumes are shown, yellow arrows highlight example structure best resolved in RLN compared to RLN-a and RLD. Scale bars: 3 µm.

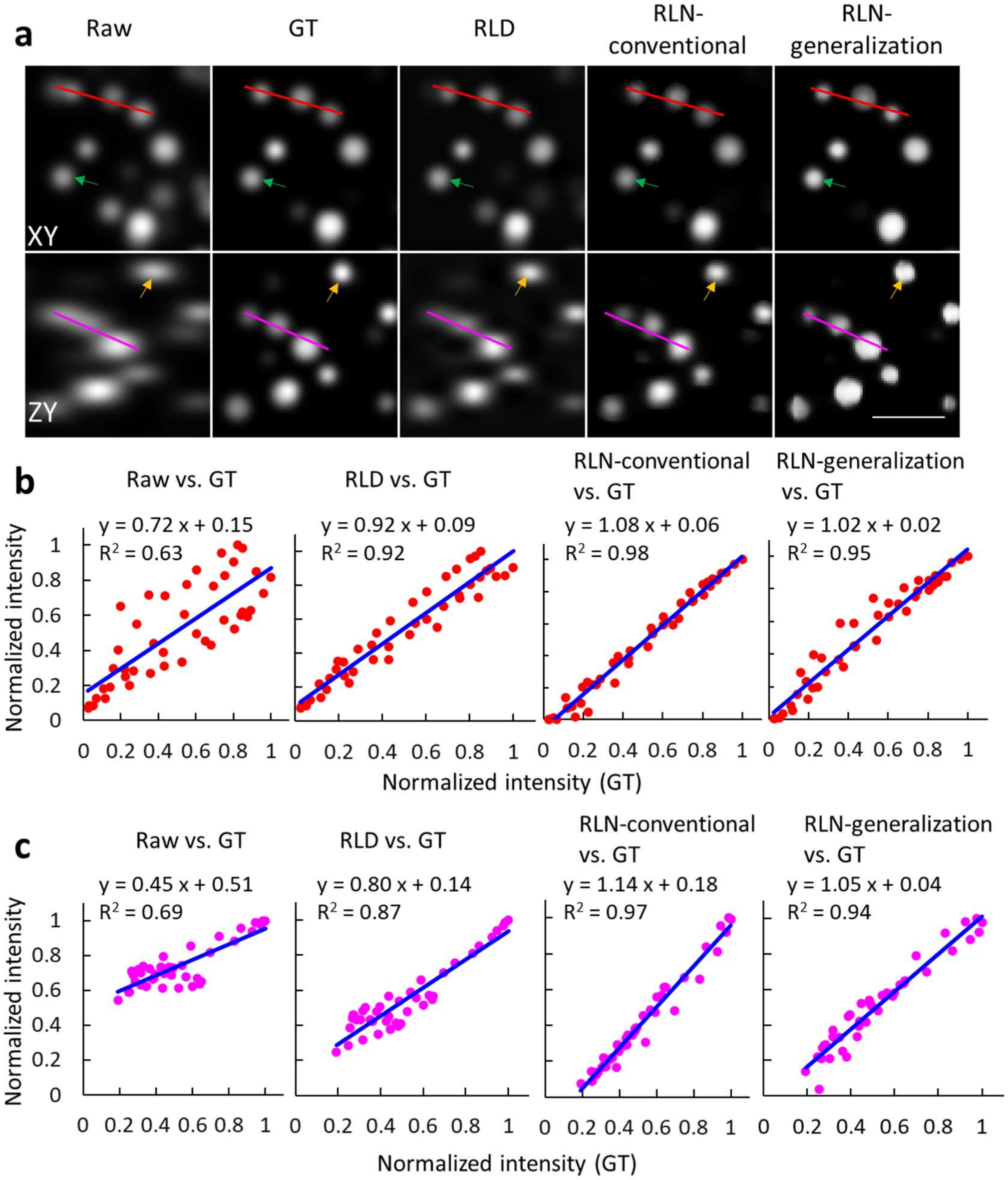

Supplementary Fig. 5, RLN performance on simulated bead samples, comparing RLD, conventional testing, and generalization. a) Raw image, ground truth (GT), RLD result, and RLN predictions in lateral (top) and axial (bottom) views. The model for generalization was trained with phantom objects consisting of mixed dots, solid spheres, and ellipsoidal surfaces, whereas the model for the conventional test used the same type of training data as the test data (simulated beads). Although RLN always outperforms RLD, the generalization result slightly distorts and sharpens bead shapes compared to the ground truth and conventional testing result (green and yellow arrows). Scale bar: 5 µm. **b)** Normalized intensity of raw, RLD, and RLN predictions (y axis) vs. normalized intensity of ground truth (x axis) taken along the red line shown in the lateral view in **a)**. The scattered red dots indicate pixel intensities, the solid blue line is the linear fit to the data, and the insets display the fitting equation and the square of the correlation coefficient (R^2). **c)** as in **b)**, but for the magenta line shown in the axial view in **a)**. These data suggest that the RLN linearity is better than RLD.

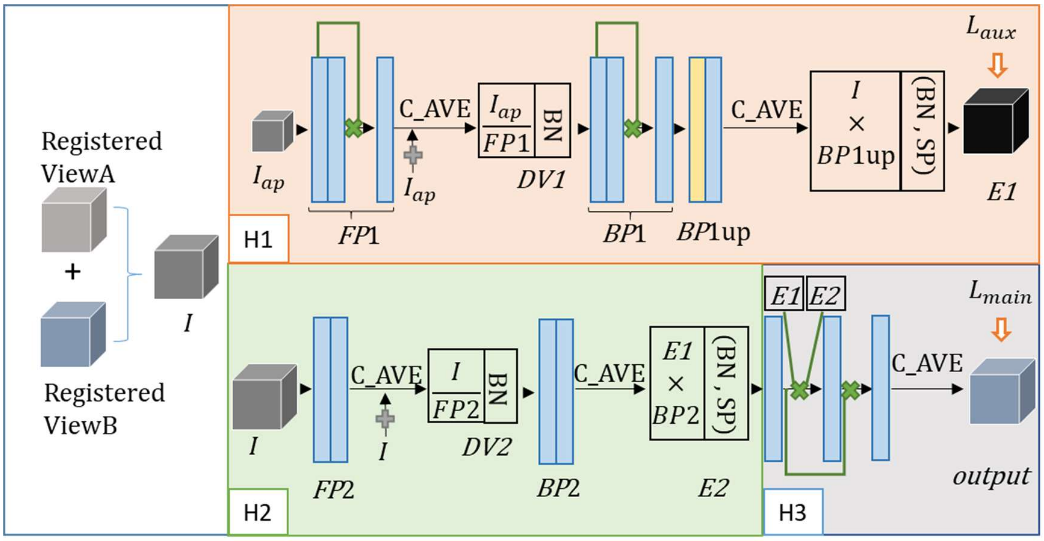

Supplementary Fig. 6, Dual-input RLN. The registered dual views are merged by averaging before applying the network.

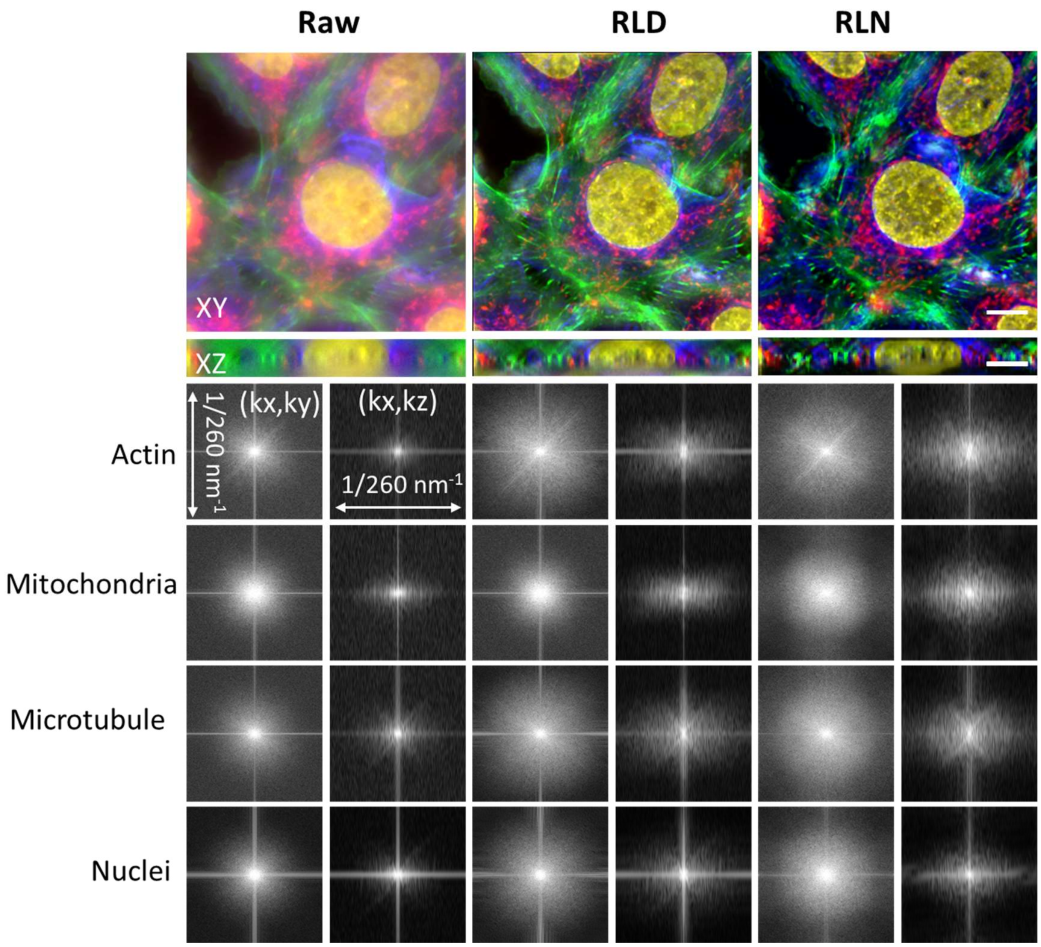

Supplementary Fig. 7, **Four color lateral and axial maximum intensity projections and Fourier spectra of a fixed U2OS cell.** Images were acquired by widefield microscopy; here raw input, RLD, and RLN predictions based on a model trained on the synthetic mixed structures are compared. See also **Fig. 5a-c**. Red: mitochondria immunolabeled with anti-Tomm20 primary antibody and donkey α-rabbit-Alexa-488 secondary; green: actin stained with phalloidin-Alexa Fluor 647; Blue: tubulin immunolabeled with mouse-α-Tubulin primary and goat α-mouse-Alexa-568 secondary; yellow: nuclei stained with DAPI. Images and Fourier spectra in axial and lateral views indicate that RLD better recovers resolution than RLD. Scale bars: 20 µm.

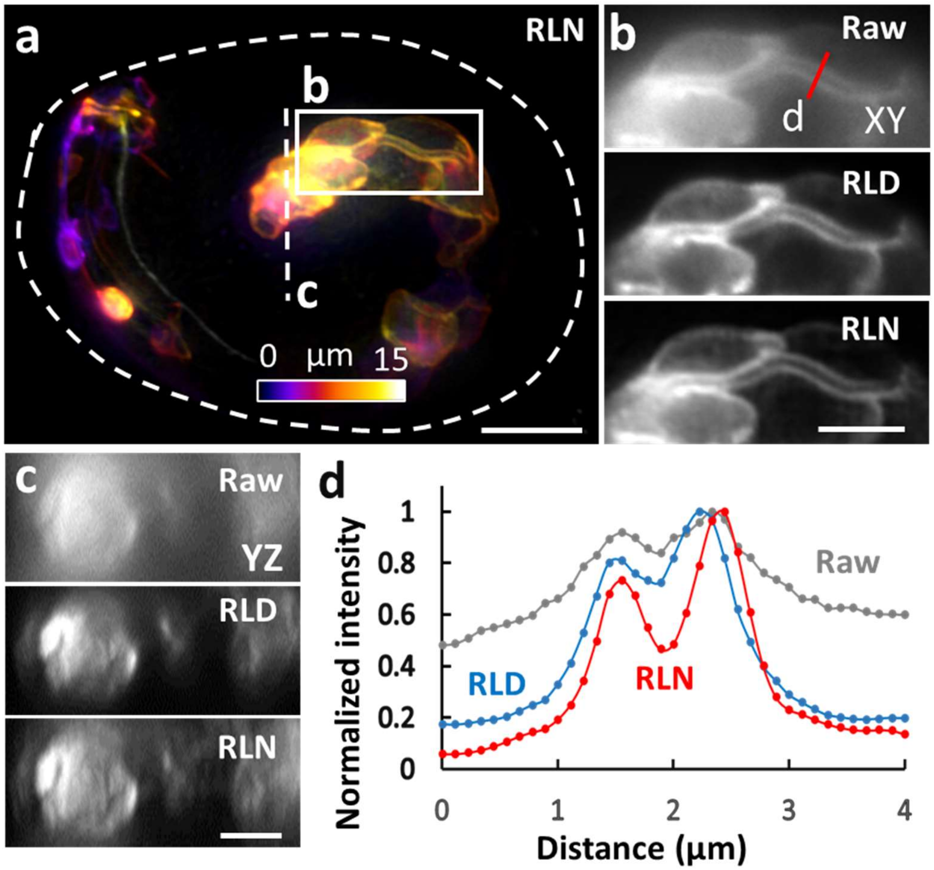

Supplementary Fig. 8, **RLN trained with synthetic mixed structures outperforms direct RLD on a *C. elegans* embryo stack. a)** Depth-coded reconstruction of *C. elegans* embryo expressing ttx-3B-GFP, acquired by widefield microscopy, and predicted by RLN based on a model trained on synthetic mixed structures. Dashed line indicates the embryo shape. See also **Fig. 5d-f**. **b, c)** Higher magnification of white rectangle and dashed line in **a)**, comparing the raw input, RLD, and RLN prediction, highlighting the membranes of individual gut cells in lateral **b)** and axial views **c)**. **d)** Line profiles of the red line shown in **b)**, indicating that RLN better resolves the membrane structure than RLD. Scale bars: **a)** 10 µm, **b, c)** 5 µm.

Supplementary table 1, Summary of RLN training and testing datasets.

	Figures	Training dataset	Ground truth	Training data size (Volume size, Volume #)	Testing dataset	Testing data size (Volume size, Volume #)
Deconvolution with simulated data	**Extended Data Fig. 2, Supplementary Figs. 2a, 4**	Phantom objects consisting of dots, solid spheres, and ellipsoidal surfaces.<br>Blurring PSF: 0.8/0.8 NA diSPIM	Phantom objects	128 x 128 x 128<br>100 pairs	Same type as training dataset	128 x 128 x 128<br>9
	**Supplementary Fig. 5**	Simulated spherical beads	Spherical beads	128 x 128 x 128<br>100 pairs	Same type as training dataset	256 x 256 x 256<br>3
Generalization with simulated data	**Fig. 1d Supplementary Figs. 2b-f, 5**	Phantom objects consisting of dots, solid spheres, and ellipsoidal surfaces.<br>Blurring PSF: 0.8/0.8 NA diSPIM	Phantom objects	128 x 128 x 128,<br>100 pairs	Simulated spherical beads	256 x 256 x 256<br>3
	**Fig. 1e Supplementary Fig. 3**				Human brain phantom	256 x 256 x 128<br>1
Deconvolution with biological images	**Fig. 2a-c**	U2OS cell, mitochondria, acquired with 0.8/0.8 NA diSPIM	Dual-view deconvolved light-sheet data	349 x 512 x 90<br>12 pairs	Same type as training dataset	349 x 512 x 90<br>50
	**Supplementary Video 1**	U2OS cell, mitochondria, acquired with 0.8/0.8 NA diSPIM	Dual-view deconvolved light-sheet data	349 x 512 x 90<br>12 pairs		284 x 382 x 100<br>200
	**Fig. 2d-e**	Mouse brain neurites acquired by cleared' tissue 0.4/0.4 NA diSPIM	Dual-view deconvolved light-sheet data	128 x 128 x 128<br>12 pairs		228 x 228 x 1218<br>25
	**Fig. 2g-i, Extended Data Fig. 3b, c Supplementary Video 2**	Cleared brain tissue slab expressing tdTomato in axons, acquired with 0.7/0.7 NA cleared tissue diSPIM	Dual-view deconvolved light-sheet data	256 x 256 x 256<br>40 pairs		1500 x 1500 x 42<br>900
	**Fig. 1b, Extended Data Figs. 4c-f, 5a**	*C. elegans* membrane acquired by 0.8/0.8 NA diSPIM	Dual-view deconvolved light-sheet data	290 x 364 x 277<br>12 pairs		290 x 364 x 277<br>9
	**Extended Data Figs. 4a, b, 5b**	*C. elegans* nuclei acquired by 0.8/0.8 NA diSPIM	Dual-view deconvolved light-sheet data	240 x 360 x 246<br>12 pairs		240 x 360 x 246<br>9
	**Supplementary Video 3**	*C. elegans* nuclei acquired by 0.8/0.8 NA diSPIM	Dual-view deconvolved light-sheet data	240 x 360 x 246<br>12 pairs		240 x 360 x 246<br>291
	**Fig. 3a-c, Extended Data Figs. 6a-c, 8b-e**	U2OS cell ER, mitochondria, lysosome, and Golgi acquired by iSIM	High SNR deconvolved iSIM data	512 x 512 x 24<br>120 pairs		1902 x 1550 x 20 (ER)<br>1920 x 1550 x 32 (Mito)<br>1920 x 1550 x 14 (Lyso)<br>1920 x 1550 x 20 (Golgi)<br>6
	**Extended Data Fig. 4g**	*C. elegans* embryos expressing	Deconvolution	338 x 310 x 338		338 x 310 x 338

		GCaMP3 from a myo-3 promoter acquired by reflective diSPIM	based on spatially variant PSF	26 pairs		12
	**Extended Data Fig. 9**	U2OS cells, mitochondrial membrane acquired by confocal microscopy	Super-resolution STED data	256 x 256 x 16<br>25 pairs		256 x 256 x 16<br>8
	**Extended Data Fig. 10a**	Jurkat cells expressing EMTB-3XGFP acquired by widefield microscope	Super-resolution 3D SIM data	1024 x 1024 x 32<br>46 pairs		1024x 1024 x 32<br>12
Generalization in biological images	**Fig. 3a-b, Extended Data Figs. 6, 7a-b**	U2OS cell ER acquired by iSIM	High SNR deconvolved iSIM data	512 x 512 x 24<br>120 pairs	U2OS cell mitochondria, lysosome, and Golgi collected by iSIM	1920 x 1550 x 32 (Mito)<br>1920 x 1550 x 14 (Lyso)<br>1920 x 1550 x 20 (Golgi)<br>6
	**Fig. 3c**	U2OS cell mitochondria acquired by iSIM	High SNR deconvolved iSIM data	512 x 512 x 24<br>120 pairs	U2OS cell ER collected by iSIM	1920 x 1550 x 20 (ER)<br>6
	**Extended Data Fig. 7d-e**	Phantom objects consisting of dots, solid spheres, and ellipsoidal surfaces. Blurring PSF: iSIM PSF	Phantom objects	128 x 128 x 128<br>100 pairs	U2OS cell lysosome acquired by iSIM	1920 x 1550 x 14<br>6
	**Fig. 3e**	Phantom objects consisting of dots, solid spheres, and ellipsoidal surfaces. Blurring PSF: iSIM PSF	Phantom objects		U2OS cell ER collected by iSIM	1920 x 1550 x 20<br>6
	**Fig. 4**	Phantom objects consisting of dots, solid spheres, and ellipsoidal surfaces. Blurring PSF: widefield PSF (63X, NA = 1.40 oil lens)	Phantom objects		U2OS cells stained with Alexa Fluor 568 - Phalloidin and Cos-7 cell immunolabeled with primary mouse anti-Nup clone Mab414 and goat-anti-mouse IgG secondary antibody conjugated with Star635P acquired by widefield microscopy	2048 x 2048 x 61<br>12
	**Fig. 5a-c Supplementary Fig.7**	Phantom objects consisting of dots, solid spheres, and ellipsoidal surfaces.<br>Blurring PSF: widefield PSF (60X, NA=1.42 oil immerse)	Phantom objects		Fixed U2OS cell acquired by widefield microscopy	512 x512 x37<br>4
	**Fig. 5d-f Supplementary Fig.8**	Phantom objects consisting of dots, solid spheres, and ellipsoidal surfaces.<br>Blurring PSF: widefield PSF (100X, NA = 1.35 silicon oil lens)	Phantom objects		Widefield *C.* elegans embryos acquired by widefield microscopy	1200 x 1200 x 201<br>6
	**Extended Data Fig. 5a**	Phantom objects consisting of dots, solid spheres, and ellipsoidal	Phantom objects		*C. elegans* membrane imaged with 0.8/0.8 NA diSPIM	290 x 364 x 277<br>1

	**Extended Data Fig. 5b**	surfaces. Blurring PSF: 0.8/0.8 NA diSPIM			*C. elegans* nuclei imaged with 0.8/0.8 NA diSPIM	240 x 360 x 246<br>1
	**Extended Data Fig. 10b**	Jurkat cells expressing EMTB-3XGFP acquired by widefield microscope	Super-resolution 3D SIM data	1024x 1024 x 32<br>46 pairs	U2OS cells expressing Lamp1-EGFP acquired by widefield microscopy	1024 x 1024 x 32<br>12

Supplementary Table 2, SSIM and PSNR values (means +/- standard deviations from N measurements) comparing RLN, RLD, CARE, RCAN, DDN, and Thunder. RLN always provides the best performance, i.e., highest SSIM and PSNR.

Figure	Sample		RLN	RLD	CARE	RCAN	DDN	Thunder	N
**Fig. 1d**	Simulated spherical beads	SSIM	0.97 ± 0.01	/	0.93 ± 0.01	0.90 ± 0.02	0.93 ± 0.02	/	12
		PSNR	35.7 ± 0.69	/	33.6 ± 0.83	32.9 ± 0.56	33.9 ± 0.45	/	12
**Fig. 1e, Supplementary Fig. 3g**	Brain phantom	SSIM	0.91 ± 0.02	/	0.83 ± 0.02	0.81 ± 0.03	0.88 ± 0.02	/	131
		PSNR	30.7 ± 0.43	/	28.9 ± 0.22	26.7 ± 0.48	27.1 ± 0.33	/	131
**Fig. 2b-c**	U2OS cells, mitochondrial label, collected with diSPIM	SSIM	0.82 ± 0.02	/	0.66 ± 0.08	0.71 ± 0.06	0.63 ± 0.03	/	50
		PSNR	26.9 ± 0.87	/	23.1 ± 2.57	25.5 ± 1.02	23.6 ± 0.60	/	50
**Fig. 2e**	Mouse brain neurites collected by cleared tissue diSPIM	SSIM	0.82 ± 0.09	/	0.74 ± 0.12	0.65 ± 0.09	0.67 ± 0.10	/	91
		PSNR	30.4 ± 3.77	/	29. 8 ± 2.59	26.7 ± 4.02	28.3 ± 1.71	/	91
**Fig. 3e**	U2OS cells, ER label, collected with ISIM, using synthetic mixed structure model	SSIM	0.69 ± 0.04	/	0.58 ± 0.04	0.59 ± 0.05	0.61 ± 0.04	/	6
		PSNR	22.0 ± 1.76	/	19.9 ± 1.73	20.1 ± 1.18	20.5 ± 1.42	/	6
**Fig. 4b-c**	fixed Cos-7 cell immunolabeled with primary mouse anti-Nup clone Mab414 and goat-anti-mouse IgG secondary antibody conjugated with Star635P, collected by	SSIM	0.86 ± 0.01	0.79 ± 0.02	/	/	/	0.80 ± 0.04	4
		PSNR	37.5 ± 0.30	36.7 ± 0.50	/	/	/	36.7 ± 0.40	4

	widefield microscopy								
**Fig. 4a**	fixed U2OS cells stained with Alexa Fluor 568 :Phalloidin, collected by widefield microscopy	SSIM	0.73 ± 0.02	0.67 ± 0.03	/	/	/	0.63 ± 0.05	4
		PSNR	30.9 ± 0.9	30.0 ± 0.7	/	/	/	30.0 ± 0.7	4
**Supplementary Fig. 4**	synthetic mixed structure, No noise	SSIM	0.95 ± 0.03	0.70 ± 0.05	/	/	/	/	9
		PSNR	28.7 ± 2.70	19.9 ± 1.58	/	/	/	/	9
	synthetic mixed structure, Poisson noise	SSIM	0.92 ± 0.03	0.59 ± 0.05	/	/	/	/	9
		PSNR	26.3 ± 2.18	19.5 ± 1.58	/	/	/	/	9
	synthetic mixed structure, Gaussian noise	SSIM	0.89 ± 0.03	0.65 ± 0.04	/	/	/	/	9
		PSNR	25.4 ± 2.06	19.1 ± 1.31	/	/	/	/	9
	synthetic mixed structure, mixed Poisson and Gaussian noise	SSIM	0.87 ± 0.03	0.57 ± 0.04	/	/	/	/	9
		PSNR	24.7 ± 1.86	18.2 ± 1.41	/	/	/	/	9
**Extended Data Fig. 7f**	U2OS cells, lysosome label, collected with ISIM, using synthetic mixed structure model	SSIM	0.72 ± 0.03	/	0.65 ± 0.02	0.66 ± 0.03	0.50 ± 0.03	/	6
		PSNR	27.0 ± 1.34	/	23.3 ± 1.18	22.3 ± 1.38	19.1 ± 1.63	/	6
**Extended Data Fig. 7c**	U2OS cells, mitochondrial label, collected with ISIM, using model trained on ER	SSIM	0.97 ± 0.02	/	0.94 ± 0.02	0.96 ± 0.02	0.95 ± 0.02	/	6
		PSNR	40.3 ± 3.43	/	32.1 ± 5.52	36.3 ± 4.81	35.4 ± 2.91	/	6
**Extended Data Fig. 9**	U2OS cells immunolabeled with a primary antibody against Tomm20 and an anti-rabbit secondary antibody conjugated with Alexa Fluor 594, acquired with confocal microscope	SSIM	0.75 ± 0.04	/	/	0.72 ± 0.04	/	/	10
		PSNR	28.1 ± 1.21	/	/	26.1 ± 1.15	/	/	10
**Extended Data Fig. 10c**	Jurkat cells expressing EMTB-3XGFP, collected by widefield microscopy	SSIM	0.92 ± 0.04	0.84 ± 0.04	/	/	/	/	12
		PSNR	38.2 ± 1.73	31.3 ± 1.25	/	/	/	/	12

**Extended Data Fig. 10d**	U2OS cells expressing EGFP-Lamp1, collected by widefield microscopy	SSIM	0.83 ± 0.06	0.73 ± 0.07	/	/	/	/	12
		PSNR	33.1 ± 3.29	30.6 ± 3.66	/	/	/	/	12

Supplementary Table 3, Neural network parameters used in RLN training (RLN-a used the same parameters) and iteration number used in RLD.

	Deconvolution ability with simulated data	Generalization ability with simulated data	Deconvolution ability in biological images								Generalization ability in biological images			
Figure	**Extended Data Fig. 2, Supplementary Figs. 4, 5**	**Figs. 1d, 1e-f Supplementary Figs. 2, 3, 5**	**Fig. 2a-c**	**Fig. 2d, e, g-i, Extended Data Fig. 3b, c**	**Fig. 3a-c, Extended Data Figs. 6a, b, 8**	**Fig. 1b, Extended Data Figs. 4a-e, 5**		**Extended Data Fig. 4g**		**Extended Data Figs. 9, 10**	**Fig. 3a-c, Extended Data Figs. 6a, b, 7a-c**	**Fig. 3e, Extended Data Fig. 7d-f**	**Fig.4, Fig. 5a-c Fig. 5d-f Supplementary Figs. 7, 8**	**Extended Data Fig. 5**
Block size	64 x 64 x 64	64 x 64 x 64	64 x 64 x 64	64 x 64 x 64	24 x 64 x 64	64 x 64 x 64		338 x 20 x 338		64 x 64 x 64	24 x 64 x 64	64 x 64 x 64	64 x 64 x 64	64 x 64 x 64
						single	dual	single	dual					
Training steps per epoch	100	100	120	120	120	120	120	130	130	46/25	120	100	100	100
Number of epochs	200	200	200	300	500	500	500	300	300	400	200	200	500	500
Starting learning rate	0.025	0.02	0.02	0.025	0.015	0.03	0.02	0.025	0.025	0.025	0.015	0.02	0.025	0.025
Decay rate	0.9	0.95	0.95	0.97	0.98	0.9	0.95	0.985	0.985	0.985	0.95	0.95	0.95	0.95
Decay step	200	500	500	250	500	500	500	390	390	390	200	500	1000	500
Training time	2-3h	2-3h	2-3h	3-4h	3-4h	4-5h	4-5h	~8h	~8h	2-3h	2-3h	2-3h	3-4h	3-4h
RLD iteration #	Mixed structure:40	Beads:10 brain:20	/	1 (unmatched back projector)	40	Membrane :5 Nuclei:10	/	2 (unmatched backprojector)		40 for **Extended Data Fig. 10**	/	/	100	Membrane:5 Nuclei:10

Supplementary Table 4, Parameters used in the training of CARE, RCAN and DDN neural networks.

	Generalization ability on simulated data			Deconvolution ability on biological samples				Generalization ability on biological samples					
Figure	**Fig, 1d, Fig. 1f, Supplementary Fig. 3g**			**Fig. 2b, c, e**			**Extended Data Fig. 8**	**Fig. 3e, Extended Data Fig. 7c**			**Extended Data Fig. 7f**		
Network	CARE	RCAN	DDN	CARE	RCAN	DDN	RCAN	CARE	RCAN	DDN	CARE	RCAN	DDN
Training steps per epoch	100	100	100	120	120	120	120	100	100	100	120	120	120
Epoch number	250	200	200	400	200	200	400	400	200	200	200	200	200
Learning rate	0.0004	0.0004	0.004	0.0004	0.0004	0.004	0.0004	0.0004	0.0004	0.002	0.0004	0.0004	0.002
Decay rate	0.9	/	0.985	0.9	/	0.985	/	0.9	/	0.985	0.9	/	0.985
Decay step	500	/	600	500	/	600	/	500	/	600	500	/	480
Training time	2-3h	6-7h	2-3h	3-4h	6-7h	2-3h	12-13h	3-4h	6-7h	2-3h	2-3h	6-7h	2-3h
