# Supplementary material for: Incorporating the image formation process into deep learning improves network performance
Source: Nat Methods. 2022 Oct 31;19(11):1427–37. doi: 10.1038/s41592-022-01652-7 (PMC9636023; doi:10.1038/s41592-022-01652-7)
Supplement: Supplementary file 2 — Reporting Summary [file 41592_2022_1652_MOESM2_ESM.pdf]

Corresponding author(s): Yicong Wu

Last updated by author(s): 08/29/2022

Reporting Summary

Nature Portfolio wishes to improve the reproducibility of the work that we publish. This form provides structure for consistency and transparency in reporting. For further information on Nature Portfolio policies, see our Editorial Policies and the Editorial Policy Checklist.

Statistics

For all statistical analyses, confirm that the following items are present in the figure legend, table legend, main text, or Methods section.

n/a	Confirmed	
☐	☒	The exact sample size (*n*) for each experimental group/condition, given as a discrete number and unit of measurement
☐	☒	A statement on whether measurements were taken from distinct samples or whether the same sample was measured repeatedly
☒	☐	The statistical test(s) used AND whether they are one- or two-sided<br>*Only common tests should be described solely by name; describe more complex techniques in the Methods section.*
☒	☐	A description of all covariates tested
☒	☐	A description of any assumptions or corrections, such as tests of normality and adjustment for multiple comparisons
☐	☒	A full description of the statistical parameters including central tendency (e.g. means) or other basic estimates (e.g. regression coefficient) AND variation (e.g. standard deviation) or associated estimates of uncertainty (e.g. confidence intervals)
☒	☐	For null hypothesis testing, the test statistic (e.g. *F*, *t*, *r*) with confidence intervals, effect sizes, degrees of freedom and *P* value noted<br>*Give P values as exact values whenever suitable.*
☒	☐	For Bayesian analysis, information on the choice of priors and Markov chain Monte Carlo settings
☒	☐	For hierarchical and complex designs, identification of the appropriate level for tests and full reporting of outcomes
☒	☐	Estimates of effect sizes (e.g. Cohen's *d*, Pearson's *r*), indicating how they were calculated

Our web collection on statistics for biologists contains articles on many of the points above.

Software and code

Policy information about availability of computer code

Data collection	Most synthetic images were generated with MATLAB 2019b, and the code is publicly available at https://github.com/MeatyPlus/Richardson-Lucy-Net. Experimental images were collected with home-built microscopes (wide-field, light-sheet, and super resolution) described in the Methods section of our paper. DiSPIM acquisition software was a plugin in Micromanager 1.4, and can be downloaded from http://dispim.org/software/micro-manager. Other microscope acquisition code was written in Python 3.7.0 and MATLAB 2019b, and is available upon request.
Data analysis	Richardson-lucy Network and Richardson-Lucy deconvolution algorithm is publicly available at https://github.com/MeatyPlus/Richardson-Lucy-Net and https://github.com/eguomin/regDeconProject/tree/master/WBDeconvolution. Other analysis code (e.g., calculation of SSIM, PSNR, SNR) was written in MATLAB 2019a, and is available upon request.

For manuscripts utilizing custom algorithms or software that are central to the research but not yet described in published literature, software must be made available to editors and reviewers. We strongly encourage code deposition in a community repository (e.g. GitHub). See the Nature Portfolio guidelines for submitting code & software for further information.

Data

Policy information about availability of data

All manuscripts must include a data availability statement. This statement should provide the following information, where applicable:

- Accession codes, unique identifiers, or web links for publicly available datasets
- A description of any restrictions on data availability
- For clinical datasets or third party data, please ensure that the statement adheres to our policy

The data that support the findings of this study are included in the Extended Data Figures and Supplementary Videos, with some representative source data for the

main figures (Figs. 1d, 2a, 2d, 3c, 4b, 5a) publicly available at https://zenodo.org/record/7023909#.YwlQI3HMJaR. The 3D human brain phantom can be downloaded from the Zubal Phantom website (http://noodle.med.yale.edu/zubal/data.htm). Other datasets are available from the corresponding author upon reasonable request.

Field-specific reporting

Please select the one below that is the best fit for your research. If you are not sure, read the appropriate sections before making your selection.

☒ Life sciences ☐ Behavioural & social sciences ☐ Ecological, evolutionary & environmental sciences

For a reference copy of the document with all sections, see nature.com/documents/nr-reporting-summary-flat.pdf

Life sciences study design

All studies must disclose on these points even when the disclosure is negative.

Sample size	Statistical results (mean +/- standard deviation) of resolution, SSIM and PSNR were obtained from N = 4-50 volumes or 80-131 lateral or axial slices for a single volume, which are typical sample sizes for imaging analysis [1]. For example, 12 volumes of beads data (Fig. 1d) and 50 volumes of mitochondria data (Fig. 2a) were used to compare the SSIM/PSNR between ground truth (GT) and predictions from RLN, CARE, RCAN and DDN. With this sample size, the improvement of RLN performance over other networks is obvious. For training neural networks, we used 12-120 pairs of volumes with different voxel sizes, and validated that the sample size is appropriate as the trained neural network models are not overfitting or underfitting [2].<br><br>[1] Raju Tomer, et al., “Quantitative high-speed imaging of entire developing embryos with simultaneous multiview light-sheet microscopy”, Nature Methods, 9, 755-763 (2012).<br>[2] https://docs.aws.amazon.com/machine-learning/latest/dg/model-fit-underfitting-vs-overfitting.html
Data exclusions	No data were excluded from the analysis.
Replication	The reproducibility of the experimental findings (i.e., the deconvolution and generalization of the neural network) was verified by calculating SSIM and PSNR on distinct simulated phantoms, fixed and live samples with sample size N = 4-50. Time-lapse imaging experiments were repeated at least 4 times, with similar results obtained each time. All attempts at replication were successful.
Randomization	In this study, samples were not allocated into different experimental groups.
Blinding	The investigators were not blinded to group allocation during data collection and data analysis. Blinding is commonly performed in clinical studies, where information is withheld from participants to prevent it from influencing the outcome of a trial. We don't think blinding is relevant in this study, as the same quantitative measures (e.g., SSIM, PSNR) were applied to all network outputs and used to assess network performance.

Reporting for specific materials, systems and methods

We require information from authors about some types of materials, experimental systems and methods used in many studies. Here, indicate whether each material, system or method listed is relevant to your study. If you are not sure if a list item applies to your research, read the appropriate section before selecting a response.

Materials & experimental systems

n/a	Involved in the study	
☐	☒	Antibodies
☐	☒	Eukaryotic cell lines
☒	☐	Palaeontology and archaeology
☐	☒	Animals and other organisms
☒	☐	Human research participants
☒	☐	Clinical data
☒	☐	Dual use research of concern

Methods

n/a	Involved in the study	
☒	☐	ChIP-seq
☒	☐	Flow cytometry
☒	☐	MRI-based neuroimaging

Antibodies

Antibodies used	Below is the full list of antibodies used, including supplier name, catalog number, clone name, lot number (if applicable), and final concentration used in the labelling. This information is now also included in the Methods section of our manuscript.<br>(1) Neurons in the mouse brain sample (Fig. 2d) were labeled with a primary antibody for tdTomato (Rabbit-anti-RFP, Rockland Antibodies and Assays, Cat. # 600-401-379, dilution 1:200) and goat-anti-rabbit Alexa Fluor 555 secondary antibody (Invitrogen, Cat. # A27039, dilution 1:100).

March 2021

(2) Nuclear pore complexes (Fig. 4b) in fixed COS-7 cells were immunolabeled with mouse-anti-nuclear pore complex primary antibody (BioLegend Cat. # 902902, clone Mab414, dilution 1:1000) and goat-anti-mouse secondary antibody conjugated with STAR 635P (Abberior, Cat. # ST635P-1001-500UG, polyclonal, dilution 1:200).
(3) Microtubules in U2OS cells (Figs. 5a-c, Supplementary Fig. 7) were labeled with mouse-anti-alpha tubulin (Thermo Fisher Scientific, Cat. # 322500, clone B-5-1-2, dilution 1:100) and goat-anti-mouse Alexa Fluor 488 (Invitrogen, Cat. # A11001, dilution 1:500).

(4) Mitochondria in U2OS cells (Figs. 5a-c, Supplementary Fig. 7) were labeled with rabbit-anti-Tomm20 (Abcam, Cat. # ab78547, 1:200) and goat-anti-rabbit Alexa Fluor 568 (Invitrogen, Cat. # A11036, dilution 1:500).

(5) Mitochondria in fixed U2OS cells (Extended Data Fig. 9) were immunolabeled with rabbit-anti-Tomm20 primary antibody (Abcam, Cat. # ab78547, dilution 1:200) and donkey-anti-rabbit secondary antibody conjugated with Alexa Fluor 594 (Jackson Immunoresearch, Cat. # 711-587-003, dilution 1:500).

(6) Jurkat T cell activating antibody coating (Extended Data Fig. 10a) was performed by incubating slides in a solution of mouse-anti-CD3 antibody (Thermo Fisher Scientific, Cat. # 14-0039-82, clone Hit3a, dilution 1:100).

Validation

These antibodies have been validated from the references below. Antibody labeling conditions are included in the Methods section.
(1) Rabbit-anti-RFP (Rockland, Cat. # 600-401-379):
(a) Vendor website: https://rockland-inc.com/store/Antibodies-to-GFP-and-Antibodies-to-RFP-600-401-379-O4L_24299.aspx
(b) Vendor statement: Polyclonal anti-RFP is designed to detect RFP and its variants. This antibody has been tested by ELISA, western blot, IF, and IHC, and is suitable for use in IP, ICC, dual RNA-FISH, iDISCO+, IEM, and FLOW. This antibody can be used to detect RFP by ELISA (sandwich or capture) for the direct binding of antigen.
(c) Reference: Roeles, Zhang C et al. A brainstem circuit for nausea suppression. Cell Rep. (2022)

(2) Mouse-anti-NUP (BioLegend Cat. # 902902)
(a) Vendor Website: biolegend.com/fr-lu/products/purified-anti-nuclear-pore-complex-proteins-antibody-11498
(b) Vendor Statement: MAb414 is a reliable general purpose monoclonal antibody which recognizes a related family of NPC proteins. This antibody is ideal for studying the morphology and composition of the nucleus and nuclear envelope. It is also useful in studying changes in the nuclear structure during mitosis and meiosis.
(c) Reference: Urbanek M el al. 2D and 3D FISH of expanded repeat RNAs in human lymphoblasts. Methods (2017)
(3) Mouse-anti-alpha-tubulin (Thermo Fisher Scientific, 322500):
(a) Vendor website: https://www.thermofisher.com/antibody/product/alpha-Tubulin-Antibody-clone-B-5-1-2-Monoclonal/32-2500
(b) Vendor Statement: N/A
(c) Reference: Graindorge et al. The Conoid Associated Motor MyoH Is Indispensable for Toxoplasma gondii Entry and Exit from Host Cells (2016)

(4) Rabbit-anti-Tomm20 (Abcam, ab78547):
(a) Vendor website: https://www.abcam.com/tomm20-antibody-mitochondrial-marker-ab78547.html
(b) Vendor Statement: Synthetic peptide corresponding to Human TOMM20 aa 100 to the C-terminus (C terminal) conjugated to keyhole limpet haemocyanin.
(c) Reference: Guo M et al. Rapid image deconvolution and multiview fusion for optical microscopy. Nat Biotechnol 38:1337-1346 (2020).

(5) Mouse-anti-CD3 primary antibody (Thermofisher, Cat. # 14-0039-82)
(a) Vendor website: https://www.thermofisher.com/antibody/product/CD3-Antibody-clone-HIT3a-Monoclonal/14-0039-82
(b) Vendor statement: The HIT3a monoclonal antibody reacts with human CD3e, a 20 kDa subunit of the TCR complex. Along with the other CD3 subunits gamma and delta, the epsilon chain is required for proper assembly, trafficking and surface expression of the TCR complex. CD3 is expressed by thymocytes in a developmentally regulated manner and by all mature T cells. Crosslinking of TCR with HIT3a initiates an intracellular biochemical pathway resulting in cellular activation and proliferation.
(c) Reference: Nguyen DX el al. Anti-TNF drives regulatory T cell expansion by paradoxically promoting membrane TNF-TNF-RII binding in rheumatoid arthritis. J Exp Med (2016)

Eukaryotic cell lines

Policy information about cell lines

Cell line source(s)

Human osteosarcoma (U2OS, ATCC HTB-96), human T lymphocyte (Jurkat E6-1, ATCC TIB-152, gift from Dr Lawrence E. Samelson, NIH), and African green monkey kidney fibroblast-like cell lines (COS-7, commercially provided by Leica-Microsystems) were used in this study.

Authentication

None of the cell lines used were authenticated.

Mycoplasma contamination

The cell lines were not tested for mycoplasma contamination.

Commonly misidentified lines
(See ICLAC register)

No commonly misidentified cell lines were used in the study.

Animals and other organisms

Policy information about studies involving animals; ARRIVE guidelines recommended for reporting animal research

Laboratory animals

Cleared mouse brain tissue sections were used in this study. The tissue sections were dissected from an 8-week old vasopressin

Laboratory animals	receptor 1B Cre X Ai9 male mouse provided by the National Institute of Mental Health (NIMH, Drs. Ted B. Usdin and Scott Young). The animal rooms were on a 12-hour light cycle, a temperature range of 70-74 °F and a humidity range of 30%-70%. Four nematode strains (BV24, od58, AQ2953, DCR6268) were also used in the study. All worms were cultivated at 20°C on nematode growth medium plates seeded with a lawn of Escherichia coli strain OP50. Embryos were dissected from gravid adults for imaging.
Wild animals	The study didn't involve wild animals.
Field-collected samples	The study didn't involve samples collected from the field.
Ethics oversight	All animal studies were performed in a manner consistent with the recommendations established by the Guide for the Care and Use of Laboratory Animals (National Institutes of Health), and all animal protocols were approved by the Animal Care and Use Committees in NIMH.

Note that full information on the approval of the study protocol must also be provided in the manuscript.
